# Commentary: The North West London Clinical Trials Alliance: efficiency and innovation in clinical trial delivery

**DOI:** 10.1186/s13063-024-08344-x

**Published:** 2024-07-29

**Authors:** Patrick Kierkegaard, Bowen Su, Richard Wong, Marta Boffito, Suki Balendra

**Affiliations:** 1 Cancer Research UK Convergence Science Centre, Imperial College London & The Institute of Cancer Research, London, UK; 2 Imperial College Healthcare NHS Trust, London, UK; 3 Chelsea and Westminster Hospital NHS Foundation Trust, London, UK; 4 NIHR Research Delivery Network, London, UK

**Keywords:** Clinical trials, Clinical Trials Alliance, COVID-19, Vaccine trials, Digital tools, Participant recruitment, Efficiency, Inclusivity, Equity, Diversity, and Inclusion (EDI), Public health

## Abstract

**Background:**

The set-up, activation, and delivery of clinical trials is pivotal for the advancement of medical science, serving as the primary mechanism through which new therapeutic interventions are validated for clinical use. Despite their critical role, the execution of these trials is often encumbered by a multitude of challenges. The North West London Clinical Trials Alliance (The Alliance) was established to address these complexities. It aims to bridge the gap between emerging scientific research and its clinical application through strategic collaborations among healthcare and research entities, thereby enhancing the regional ecosystem for clinical trials.

**Main text:**

This commentary aims to offer clarity on the fundamental insights that underlie The Alliance, providing a comprehensive understanding of its operational structure and the ecosystem it has fostered to optimise clinical trial delivery and revenue generation. The strategy employed by The Alliance centres on the cultivation of strategic partnerships across a broad spectrum of stakeholders. This approach addresses key operational challenges in clinical trial management, facilitating improvements in the development, setup, activation, and recruitment stages. Notably, The Alliance has reduced the average time to initiate trials to 19 days, compared to the standard 75 days typically observed for commercial setups in North West London. The effectiveness of The Alliance’s framework was notably demonstrated during the COVID-19 pandemic, particularly with the expedited recruitment performance in the Janssen COVID-19 vaccine study conducted at Charing Cross Hospital. This instance highlighted the Alliance’s capability to meet and exceed recruitment targets promptly while maintaining diversity within study cohorts. Additionally, The Alliance has effectively harnessed digital technology and infrastructure, enhancing its attractiveness to commercially funded studies and illustrating a sustainable model for clinical trial financing and execution.

**Conclusion:**

The North West London Clinical Trials Alliance represents a strategic response to the conventional challenges faced in clinical trial management, emphasising the importance of cross-sectoral collaboration and resource optimisation. Its efforts, particularly highlighted by its response to the COVID-19 pandemic, provide a case study in enhancing trial delivery and efficiency with significant implications for both regional and global clinical trials research communities.

## Background

Clinical trials are vital for generating evidence that bridges the gap between scientific discoveries and therapeutic standards of care, with the goal of improving health outcomes. The success of a clinical trial heavily depends on the study’s start-up time, the time it takes to activate the trial, and the time it takes to reach the target number of participant recruits [1, 2]. Nevertheless, the start-up and activation of clinical trials is operationally complex and frequently obstructed by systemic inefficiencies, leading to notable delays and operational difficulties [3]. Significant financial implications arise from delays and compromise the scientific validity of trials [4, 5]. Some studies have emphasised the need to enhance operational efficiencies and to improve processes to overcome these challenges [6, 7]. Other studies have discussed the advantages of forming networks or alliances to promote partnerships and collaborations between different research entities to improve research capabilities and capacity, sharing of resources, and generating revenue [8–11].

In response to the recognised benefits of clinical trial networks and alliances, and the imperative to enhance operational efficiencies and processes, the North West London Clinical Trials Alliance (henceforth referred to as ‘The Alliance’) was established. Its primary aim was to develop an ecosystem to optimise trial development, set-up, activation, and participant accrual for both commercial and non-commercially sponsored studies within the region. This necessitated the development of a framework designed to facilitate strategic collaborations across various healthcare and research organisations in North West London. A significant aspect of this framework included the establishment of a partnership pipeline with life sciences companies and the utilisation of digital technologies, alongside existing and purposefully designed facilities. This approach sought to increase trial capacity, improve operational adaptability, and enhance the inclusivity of participant recruitment. Emphasis was placed on attracting a larger number of commercially funded studies to broaden the scope of clinical research in the area. The underlying aim was to facilitate advancements in medical science by streamlining clinical trial processes and fostering collaborative efforts among healthcare and research entities.

Amid the COVID-19 pandemic, The Alliance’s efforts to expedite trial setup and ensure participant diversity were put to the test. One instance highlighting these efforts was the facilitation of the Janssen COVID-19 vaccine study at Charing Cross Hospital [12]. This trial achieved its recruitment target by enrolling 400 participants within an 8-week period, thereby exceeding initial recruitment timelines. Furthermore, the study achieved notable success in participant diversity, with 15.3% of enrolees coming from minority ethnic backgrounds (compared to the 4.4% average for this study in the UK) [13]. This accomplishment illustrates The Alliance’s contribution to improving the inclusivity and efficiency of clinical trial recruitment and participation, reflecting its commitment to enhancing the regional clinical trials ecosystem. This experience reflects part of the portfolio of trial studies highlighting the efficacy of The Alliance’s ecosystem, positioning it as a viable and scalable solution for improving the delivery and efficiency of early and late-phase trials.

Given this context, this commentary aims to offer clarity on the fundamental insights that underlie the Alliance, aiming to provide a comprehensive understanding of its operational structure and the ecosystem it has fostered to optimise clinical trial delivery and revenue generation. The intention is to share this knowledge with key decision-makers who are keenly interested in contributing to the development of local and regional clinical trial alliances and networks.

## Alliance dynamics: infrastructure and outcomes

### Strategic collaborations and network integration

Establishing strategic partnerships between diverse healthcare and research entities is the cornerstone of the Alliance. Central to this effort is a comprehensive network, composed of eight NHS Trusts and three Clinical Research Facilities (CRFs), which plays a pivotal role in the Alliance’s ability to successfully deliver trials. A critical component of this strategy involves deepening the engagement between the Trusts and CRFs and their partner sites, including community hospitals and local General Practitioners (GPs). Such involvement is key to improving trial accessibility and fostering a community-engaged approach to patient recruitment. This strategy has led to notable successes in recruitment outcomes, exemplified by enrolling over 1600 participants in COVID-19 vaccine trials. Notably, 15.3% of these enrolees came from minority ethnic backgrounds, reflecting the Alliance’s commitment to inclusivity. This achievement not only surpasses local diversity benchmarks but also highlights the effectiveness of these collaborative efforts in expanding trial participation.

Building on the framework established through strategic partnerships, incorporating specialty medical centres and academic institutions into the Alliance’s framework constitutes a significant development in its scope. These entities contribute specialised expertise and infrastructure essential for the support of a broad array of medical research endeavours. The impact of these integrations is clear in the Alliance’s capacity to initiate trials within an average timeframe of 19 days, as opposed to the standard 75 days typically observed for commercial set-ups in North West London.

Further extending the Alliance’s research infrastructure, the recent inclusion of ambulance services into its network has enhanced access to critical pre-hospital and emergency care data, as well as broadened the prospects for recruitment.

### Enhancing recruitment reach and streamlining the process

Integrating GPs as Participant Identification Centres within the Alliance’s structure diverges from conventional clinical trial recruitment strategies. By levering patient records, GPs have been able to efficiently identify potential trial participants. This method has significantly streamlined participant identification, allowing GPs to employ mass text messaging services to reach out to potential candidates for clinical trials. Similar to another study on GP participation and the recruitment of people to trials [14], this method capitalises on the trust already established between GPs and their communities, resulting in increased involvement in recruitment initiatives.

The methodological strategy adopted by the Alliance, integrating primary care records with expansive participant databases and registries, not only facilitated the inclusion of a diverse participant pool but also significantly bolstered England’s clinical trial recruitment efforts, by contributing to 5.6% of England’s total clinical trial participant recruitment—a figure that surpasses the 4.8% national average for commercial clinical trial participant recruitment. This high level of recruitment demonstrates enhanced efficiency by reducing the time needed to reach participant targets. The inclusion of a diverse participant pool, with 15.3% from minority ethnic backgrounds, highlights the success of strategies aimed at enhancing inclusivity. Furthermore, the ability to rapidly meet and exceed recruitment goals supports higher success rates in clinical trials by ensuring robust and representative sample sizes.

Notably, the Janssen COVID-19 vaccine study, which attracted 15.3% of its participants from minority ethnic backgrounds, and the Novavax COVID-19 vaccine trial, which enrolled 550 participants in just 5 weeks at the Chelsea and Westminster Hospital NHS Foundation Trust, serve as compelling evidence of the Alliance’s effective strategies in engaging underrepresented groups and expediting trial processes. This harmonised approach was instrumental in the successful delivery of eight COVID-19 vaccine trials, achieving a 100% target-to-time, further evidencing the efficiency and reach of the Alliance’s recruitment practices.

### Operational efficiency

The Alliance implemented protocols and systems designed to diminish administrative burdens, enhance data collection, and improve trial processes. Central to these efforts is the establishment of efficient referral mechanisms through primary care and Participant Identification Centres, aimed at streamlining participant recruitment. Additionally, a structured pre-screening and booking process supports local study teams, while an evaluative framework ensures that research sites are equipped with the facilities and infrastructure. This framework considers crucial aspects, such as adequate space and security measures, vital for the efficient execution of research. The adoption of a scripted appointments booking system with an appointment tracking method has fostered a participant-centred approach, resulting in the more effective management of participant engagement.

### Commercial engagement and impact

Since its inception, the Alliance has engaged in collaborations with 21 diverse life science companies, thereby creating 25 new avenues for clinical research across a variety of therapeutic areas. These joint efforts with life science companies to undertake commercially sponsored studies have yielded significant economic benefits, generating a notable commercial revenue of £16 million. This revenue is primarily derived from the successful execution of eight COVID-19 vaccine trials, highlighting the Alliance’s substantial economic and operational contributions to the clinical research domain.

The financial accomplishments of the Alliance not only demonstrate its capacity to draw and secure sizeable investments from the life sciences sector but also affirm its commercial appeal and viability. Such achievements resonate with industry stakeholders, contributing to the development of a sustainable research ecosystem.

## Conclusions

The knowledge and insights gained from the Alliance, characterised by strategic partnerships, technological integration, and a strong focus on diversity and efficiency, suggest a framework that could shape clinical trial practices at both regional and global levels. The measurable outcomes of the Alliance’s trial delivery, such as reaching recruitment targets and reducing setup times, suggest it can serve as a tangible framework for other regions seeking similar success.

The framework established by the Alliance places a strong emphasis on the development of partnerships among stakeholders in the community, healthcare providers, and research entities. Such collaborations are instrumental in facilitating the sharing of expertise, support from personnel, and the achievement of successful research collaborations [15]. The Alliance launched several key initiatives aimed at increasing diversity in clinical trial recruitment. These initiatives focused on innovating patient enrolment strategies through collaboration, optimising the inclusion of different populations into clinical trials. This will allow clinical research results to be applicable and therefore, benefit different communities and improve the health of the population in general.

Beyond improving the delivery and efficiency of trials, the considerable revenue derived from commercial studies through these partnerships presents a viable approach for crafting sustainable funding mechanisms within clinical trial research. By focusing on both collaboration and innovation, the Alliance exemplifies the possibility of achieving both commercial success and significant progress in clinical research methods. This strategy underscores the critical importance of adopting models that ensure economic viability alongside operational effectiveness.

At the same time, it is crucial to acknowledge that the Alliance’s initial successes, as discussed, took place during the COVID-19 pandemic—a time with a unique context that may have influenced the distribution of resources in ways that cannot be replicated today. Ongoing evaluation is crucial for organisations that aim to learn from and replicate the Alliance’s development and scalable practices. Implementing this strategy will enable a holistic grasp of the long-term consequences on research effectiveness and the economic development of the area. Ongoing monitoring and reporting will be required to assess the Alliance’s efficacy in improving clinical research practices and its ability to foster sustainable economic growth in the regional research sector.

## Data Availability

Not applicable.

## Abbreviations

EDI: Equity, Diversity, and Inclusion
GP: General Practitioner
CRFs: Clinical Research Facilities
